# Increased expression of REG3A promotes tumorigenic behavior in triple negative breast cancer cells

**DOI:** 10.1186/s13058-024-01845-2

**Published:** 2024-06-05

**Authors:** Xiaoxia Jin, Shuyun Yang, Xiaoyun Lu, Xudong Chen, Wencheng Dai

**Affiliations:** 1 Department of Pathology, Affiliated Tumor Hospital of Nantong University, No.30 North Tongyang Road, Pingchao, Nantong, 226361 Jiangsu China; 2 Department of Head and Neck Surgery, Affiliated Tumor Hospital of Nantong University, No.30 North Tongyang Road, Pingchao, Nantong, 226361 Jiangsu China

## Abstract

**Background:**

Identifying new targets in triple negative breast cancer (TNBC) remains critical. REG3A (regenerating islet-derived protein 3 A), a calcium-dependent lectin protein, was thoroughly investigated for its expression and functions in breast cancer.

**Methods:**

Bioinformatics and local tissue analyses were employed to identify REG3A expression in breast cancer. Genetic techniques were employed to modify REG3A expression, and the resulting effects on the behaviors of breast cancer cells were examined. Subcutaneous xenograft models were established to investigate the involvement of REG3A in the in vivo growth of breast cancer cells.

**Results:**

Analysis of the TCGA database uncovered increased *REG3A* levels in human breast cancer tissues. Additionally, *REG3A* mRNA and protein levels were elevated in TNBC tissues of locally treated patients, contrasting with low expression in adjacent normal tissues. In primary human TNBC cells REG3A shRNA notably hindered cell proliferation, migration, and invasion while triggering caspase-mediated apoptosis. Similarly, employing CRISPR-sgRNA for REG3A knockout showed significant anti-TNBC cell activity. Conversely, REG3A overexpression bolstered cell proliferation and migration. REG3A proved crucial for activating the Akt-mTOR cascade, as evidenced by decreased Akt-S6K1 phosphorylation upon REG3A silencing or knockout, which was reversed by REG3A overexpression. A constitutively active mutant S473D Akt1 (caAkt1) restored Akt-mTOR activation and counteracted the proliferation inhibition and apoptosis induced by REG3A knockdown in breast cancer cells. Crucially, REG3A played a key role in maintaining mTOR complex integrity. Bioinformatics identified zinc finger protein 680 (ZNF680) as a potential REG3A transcription factor. Knocking down or knocking out ZNF680 reduced REG3A expression, while its overexpression increased it in primary breast cancer cells. Additionally, enhanced binding between ZNF680 protein and the REG3A promoter was observed in breast cancer tissues and cells. In vivo, REG3A shRNA significantly inhibited primary TNBC cell xenograft growth. In REG3A-silenced xenograft tissues, reduced REG3A levels, Akt-mTOR inhibition, and activated apoptosis were evident.

**Conclusion:**

ZNF680-caused REG3A overexpression drives tumorigenesis in breast cancer possibly by stimulating Akt-mTOR activation, emerging as a promising and innovative cancer target.

**Supplementary Information:**

The online version contains supplementary material available at 10.1186/s13058-024-01845-2.

## Introduction

Breast cancer stands as the most prevalent malignancy among women globally and a leading cause of cancer-related deaths [1, 2]. Breast cancer treatment encompasses a range of modalities tailored to the cancer’s type, stage, and individual factors. Surgery often precedes other treatments like radiation therapy [3–5]. Chemotherapy and hormone therapy target cancer cells systemically or by blocking hormone receptors, while targeted therapies like HER2-targeting drugs or immunotherapy focus on specific molecular abnormalities or harness the immune system against cancer cells [3–5]. However, despite these treatment advancements, the prognosis remains unsatisfactory for individuals exhibiting drug resistance or experiencing cancer recurrence [3, 4, 6]. Additionally, therapeutic options for triple-negative breast cancer (TNBC) remain limited, contributing to a typically unfavorable prognosis [7, 8]. There is an urgent need to uncover novel signaling proteins crucial for fostering the growth of breast cancer [7, 8].

REG3A (regenerating islet-derived protein 3 A) belongs to the regenerating family proteins and functions as a calcium-dependent lectin protein [9, 10]. Its expression primarily occurs in gastrointestinal organs, such as the pancreas, stomach, and intestinal tracts and it demonstrates robust upregulation during infection or inflammation within the digestive system [10, 11]. REG3A governs the interaction between the host and microbiota [11, 12]. Studies have shown that depletion of REG3A led to increased bacterial colonization, mucosal inflammation, and activation of the adaptive immune response in mice [11, 12]. Conversely, using genetic approaches to over-express REG3A restricted bacterial infection and associated inflammation [13].

Several recent studies have suggested a potential involvement of REG3A in the onset and advancement of various human cancers. Chen and colleagues indicated that the upregulation of REG3A can facilitate gastric cancer cell proliferation by potentially modulating the JAK2-STAT3 cascade [14]. In colorectal cancer, elevated REG3A levels is important for fostering cancer cell proliferation and migration [15]. REG3A was found to elevate Akt and ERK1/2 phosphorylation while governing the base excision repair cascade [15]. Xu and colleagues observed heightened REG3A expression in pancreatic cancer cells, correlating with enhanced cancer growth potentially via the augmentation of various oncogenic gene expressions [16]. Additionally, increased REG3A expression exhibited pro-proliferative effects in pancreatic cancer cells [17]. In hepatocellular carcinoma (HCC), REG3A o knockdown resulted in reduced HCC proliferation while promoting apoptosis [18].

Interestingly, few studies have also highlighted the potential anti-cancer effects associated with REG3A. Qiu and coworkers demonstrated that REG3A overexpression triggered proliferation inhibition and induced apoptosis in colorectal cancer cells [19]. Wang et al. suggested a tumor-suppressive role for REG3A, indicating its capacity to increase DMBT1 (deleted in malignant brain tumors 1) expression in gastric cancer [20]. However, investigations into the expression and potential functions of REG3A in TNBC and other breast cancers remain scarce. The results of this current study are expected to reveal that overexpressed REG3A behaves as a pro-cancerous protein in breast cancer, positioning it as a potential and significant therapeutic target.

## Materials and methods

### Reagents and antibodies

Puromycin, serum, medium, polybrene, RNA assay-related reagents, LY294002, and caspase inhibitors, as well as cell counting kit-8 (CCK-8) reagent and Trypan blue were purchased from Sigma-Aldrich Chemicals (St. Louis, Mo). Fluorescence dyes, including EdU (5-ethynyl-2’-deoxyuridine), TUNEL (terminal deoxynucleotidyl transferase dUTP nick end labeling), and JC-1 were provided by Invitrogen Thermo-Fisher (Shanghai, China). The anti-REG3A antibody (ab202057) and anti-REG1 antibody (ab47099) were obtained from Abcam (Shanghai, China). The anti-ZNF680 antibody was from Sigma. All other antibodies were provided by Dr. Zha [21].

### Cells

MDA-231 and MCF-7 breast cancer cell lines and a non-tumorigenic epithelial cell line, MCF-10 A, were provided by Dr. Cao [22] and maintained in the described medium [22]. The protocols for the primary culture of human TNBC cells were described in an early study [22]. Fresh cancer tissues and cancer-adjacent normal mammalian epithelial tissues were freshly obtained at the time of surgery, separately, carefully under microscopy, and thoroughly washed using the described medium [22]. Fresh tissues were cut into small pieces and incubated with 0.20% (w/v) collagenase (in DMEM) for 1 h. Macrophages, vascular cells, fibroblasts, and debris were carefully removed. Individual breast cancer cells and mammary epithelial cells (“pMEC”) were thereafter pelleted, washed, and cultivated under the described complete medium [22]. The enrolled patients each provided informed-consent. The primary breast cancer cells were derived from two TNBC patients, namely “pBC-1” and “pBC-2”. These primary cancer cells were TNBC cells with *PTEN* depletion. pMEC were derived from one single patient. The protocols for using primary human cells were approved by the Ethics Committee of Nantong University (TY-B2022-0144) and were according to the Declaration of Helsinki.

### Human tissues

The breast cancer tissues and matched adjacent normal tissues were obtained from a cohort of twenty (20) primary TNBC patients. All patients were administrated at the authors’ institution, and each provided written-informed-consent. Tissues were freshly obtained at the time of surgery and subjected to quantitative real-time PCR (qRT-PCR), Western blotting, and immunohistochemistry (IHC) assays. The protocols for testing human tissues were approved by the Ethics Committee of Nantong University (TY-B2022-0144) and were in accordance with the Helsinki Declaration.

### Quantitative real-time PCR (qRT-PCR), Western blotting, and co-immunoprecipitation (Co-IP) assays

Total RNA was extracted from cells or tissues and quantified. It was then reversely transcripted to cDNA under a PrimeScript RT reagent kit (Takara Bio, Japan). The detailed protocols for qRT-PCR and data quantification were reported in other studies [23]. *GAPDH* expression was tested as the internal control and the reference gene. The detailed protocols of Western blotting assays and Co-IP (for mTOR complexes) were reported elsewhere [21, 24]. Figure S1 lists the uncropped blotting images.

### shRNA

Breast cancer cells or mammary epithelial cells were cultivated in complete medium with polybrene at 60–65% confluence. A total of six different shRNAs targeting non-overlapping sequences of *REG3A* (shREG3A-Sq1 to shREG3A-Sq6) were designed and provided by Genechem (Shanghai, China). Each was individually inserted into a lentiviral construct. The construct, together with the lentivirus envelope plasmids, were co-transfected into HEK-293 cells, generating shRNA-expressing lentivirus. Which were added to the cultured cells at MOI = 12. The virus infection lasted for 48 h. Afterwards, puromycin-containing complete medium was added to infected cells and stable cells were formed after 4–6 passages’ selection. Three different shRNAs were utilized: shREG3A-Sq2 targeting *CTGTAATGTGAGGTTACCCTATGTC*, shREG3A-Sq3 targeting *TGTTTGGTGTGCAACTCATCATG* and shREG3A-Sq6 targeting *CCCTGGTGAAGAGCATTGGTAAC*. The control cells were infected with lentiviral particles with scramble control shRNA (“shC”). The mRNA and protein expression of REG3A in the stable cells were always tested. shRNA-induced silencing of ZNF680 was done through the same procedures with different sequences, with shZNF680-Sq1 targeting *AGGCACTGACACTTTAGACATTACA* and shZNF680-Sq2 targeting *TTGACAAAGCCTTCCAATGATTGTT*.

### Gene knockout (KO)

Breast cancer cells were cultivated in complete medium with polybrene at 60–65% confluence and were infected with dCas9-expressing lentivirus (from Dr. Ling [25]), and stable cells formed after selection. The lentivirus encoding the CRISPR/Cas9-REG3A-KO puro-construct (containing sgRNA against REG3A, targeting *GTAACAGCTACTCATACGTC*, PAM *TGG*), provided by Genechem (Shanghai, China), was added to dCas9-expressing cells and stable cells were formed by puromycin treatment. Cells were then distributed into 96-well plates and subjected to *REG3A* KO screening. Finally, single stable REG3A KO (“koREG3A”) breast cancer cells were formed. The control cells were infected with lentivirus encoding the CRISPR/Cas9-empty control vector (“koC”, from Dr. Ling [25]). The CRISPR/Cas9-induced knockout of ZNF680 was done through the same procedure, with verified sgRNA targeting ZNF68 (targeting *GCCTCACCTGATAACCTGTT*, PAM *TGG*) inserted in the CRISPR/Cas9 construct.

### Gene overexpression

Breast cancer cells were cultivated in complete medium with polybrene at 60–65% confluence and were infected with lentivirus encoding the REG3A-expressing construct (Genechem) at MOI = 12. The construct contained the REG3A cDNA [NM_002580.2] sequence. The virus infection lasted for 48 h. Afterwards, puromycin-containing complete medium was added to infected cells and two stable cell selections, “oeREG3A-Slc1” and “oeREG3A-Slc2”, were formed after another 4–6 passages. REG3A expression in the stable cells was always tested. ZNF680 overexpression was done through the same procedure, with the ZNF680 cDNA (NM_001130022.2) inserted in the construct.

### Cell viability and death assays

In brief, 2, 500 cells per well were seeded onto 96-well plates and further cultivated for 96 h. Afterwards, CCK-8 reagent was added to each well and incubated for an additional 2 h. CCK-8’s optical density (OD) was measured through a microplate reader at 450 nm. Alternatively, cells were stained with Trypan blue, and “dead” cells with positive Trypan blue staining were automatically measured via a cell counter.

### Cell migration/invasion assays

The 8 μm-pore “Transwell” chambers (BD Bioscience, San Jose, CA) were utilized. For cell migration assays, breast cancer cells (10, 000 cells per well) with the designated genetic modifications were re-suspended in 200 µL serum-free medium and added to the upper chamber surface. The lower chambers were filled with complete medium. Cells were allowed to migrate for 24 h. Afterwards, migrated cells were fixed and stained. For cell invasion assays, the inserts were pre-coated with Matrigel (Sigma), and other steps were the same.

### Caspase-3 activity

In brief, the caspase-3 activities in cell lysates or xenograft tissue lysates (20 µg lysates pre-treatment) were measured via the Caspase-3 Colorimetric Assay kit (BioVision, Milpitas, CA) according to the manufacturer’s instructions.

### Fluorescence dye assays

The breast cancer cells, or mammary epithelial cells, with the designated genetic modifications, were seeded into twelve-well plates at 65–75% confluence and cultivated for designated hours. The cells were then fixed, permeabilized, washed, and incubated with the designated fluorescence dyes. After washing with PBS, the fluorescence signals were captured via a Zeiss confocal microscope, and their intensity was quantified.

### Akt1 mutation

The described breast cancer cells were first placed on six well plates at 50–60% confluence and maintained in polybrene-containing complete medium. Thereafter, cells were transduced with a lentiviral S473D constitutively-active mutant Akt1 construct (caAkt1, from Dr. Xu [26], with no tag) or the empty vector. After selection using puromycin, stable cells were formed, and caAkt1 expression was verified in the stable cells.

### siRNA

Genechem (Shanghai, China) provided validated siRNAs targeting different transcription factors (Foxl2, ZNF680, ZSCAN31, Nr1H2, and Foxo1) used at a concentration of 200 nM for individual transfection into breast cancer cells using Lipofectamine 3000. The transfection was repeated once at 24 h and terminated at 48 h. Each siRNA achieved a minimum of 70% reduction in targeted mRNA expression. Control cells were transfected with a non-sense control siRNA (siC).

### Chromatin immunoprecipitation (ChIP)

Tissue lysates or total cellular lysates, following homogenization using a homogenizer, were diluted in ChIP dilution buffer as described previously. These lysates were then subjected to immunoprecipitation using an anti-ZNF680 antibody, and ZNF680-associated DNA was subsequently eluted using protein A/G agarose (Santa Cruz Biotech) containing NaCl. The proposed REG3A promoter sequence from the JASPAR database was quantitatively evaluated via PCR (qPCR).

### Xenograft study

The female nude mice, weighing 18.2–18.9 g, were purchased from Changzhou Cavens Experimental Animal Center (Changzhou, China). The pBC-1 primary breast cancer cells, with shREG3A-Sq6 (“shREG3A”) or control shRNA (“shC”), were dissolved in serum free medium and were subcutaneously (*s.c.*) injected into the flanks of the nude mice (5 × 10 ^6^ cells per mouse). The mice body weights and tumor volumes were measured every six days [27]. The protocols were approved by the Institutional Animal Care and Use Committee (IACUC) and the Ethics Committee of Nantong University.

### Tissue immunohistochemistry (IHC) and immunofluorescence assays

For IHC staining, human tissue sections or xenograft tissue sections were permeabilized by 0.3% Triton X-100 for 15 min at room temperature and were thereafter washed. Next, tissue sections were blocked by 5% serum for 30 min. The sections were then incubated with the anti-REG3A antibody (Abcam, ab198824, 1:100) or anti-p-Akt Ser-473 antibody (Cell Signaling Tech, #31,957, 1:100) at 4 °C overnight, followed by treatment with an enzyme-labeled second antibody at room temperature for 1 h. Afterwards, tissue sections were developed [28]. For tissue immunofluorescence staining, the sections were incubated with fluorescence dyes, washed, and signals captured by a Zeiss Confocal microscope.

### Statistical analyses

All values in the study were distributed normally and were expressed as means ± standard deviation (SD). Statistical differences between multiple (three or more) groups were analyzed via one-way ANOVA plus post hoc Bonferroni test (SPSS 23.0). For comparing two specific groups, the two-tailed Student’s t-test (Excel 2013) was utilized. ***P*** values < 0.05 were statistically significant.

## Results

### REG3A expression is elevated in breast cancer tissues and cells

**Fig. 1:**
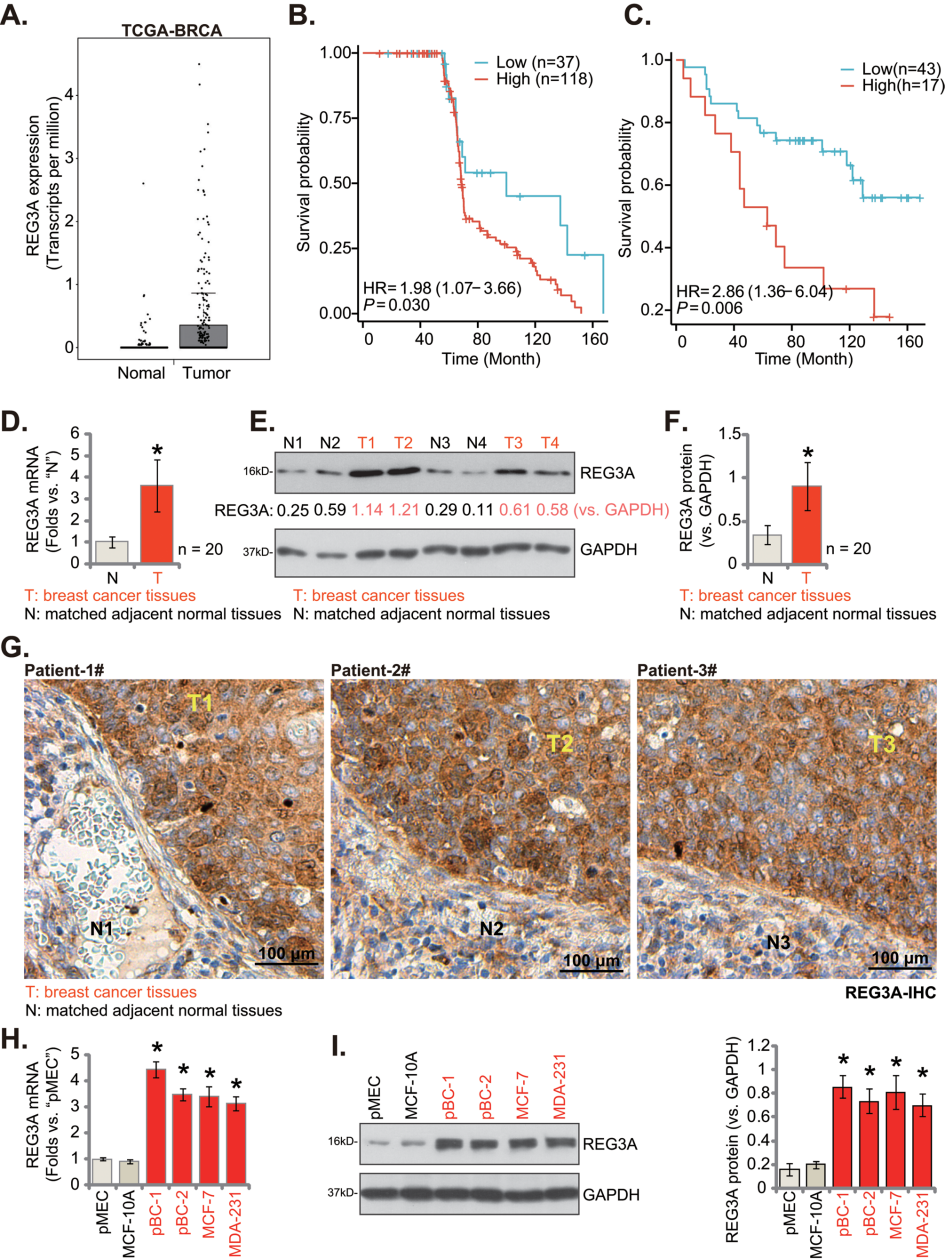
REG3A expression is elevated in breast cancer tissues and cells. The Cancer Genome Atlas (TCGA) breast cancer cohort shows *REG3A* expression (RNA-Seq) in cancer tissues (“Tumor”, *n* = 1085) and normal breast tissues (“Normal”, *n* = 291) (**A**). Data from the GEO database (GSE9893 and GSE1379) demonstrates the correlation between *REG3A* expression and patient outcomes, including overall survival (**B**) and recurrence-free survival (RFS) (**C**).Expression of listed mRNAs and proteins in twenty (*n* = 20) primary breast cancer tissues (“T”) and cancer-surrounding normal tissues (“N”) of local patients was shown (**D**-**F**). The representative REG3A immunohistochemistry (IHC) images of the described human tissues were shown (**G**). Expression of listed mRNAs and proteins in the described breast cancer cells and epithelial cells was shown (**H** and **I**). Values were mean ± standard deviation (SD). *n* = 5 for **H** and **I.** *P < 0.05 versus “Normal” tissues (**A**); *P < 0.05 versus “N” tissues (**D**-**F**); *P < 0.05 versus “pMEC” (**H** and **I**). Scale bar = 100 μm

The Cancer Genome Atlas (TCGA) breast cancer (BRCA) database was first consulted, and results showed that the number of *REG3A* transcripts in the breast cancer tissues (“Tumor”) was significantly higher than in the normal breast tissues (“Normal”) (Fig. 1A). Survival analysis was performed using breast cancer-related transcriptome data from the GEO database. The surv_cutpoint function in the survminer package was used to determine the optimal cut-off point. Data from GSE9893 indicates that higher REG3A expression is associated with poorer overall survival (Fig. 1B). Data from GSE1379 indicates that higher REG3A expression is associated with worse recurrence-free survival (RFS) (Fig. 1C).

Next, we tested the expression of REG3A in the human breast cancer tissues of local patients. The breast cancer tissues (“T”) and the matched adjacent normal tissues (“N”), from a set of twenty (20) primary patients, were obtained at the time of surgery. Fresh tissue lysates were analyzed at both the *mRNA* and protein levels. qRT-PCR assay results, Fig. 1D, demonstrated that *REG3A* mRNA expression was substantially elevated in breast cancer tissues and was about 3–4 folds of that that in normal tissues (Fig. 1D). Testing protein expression via Western blotting assays further demonstrated that REG3A protein expression was upregulated in breast cancer tissues of four representative patients (“T1/T2/T3/T4”) (Fig. 1E), whereas its expression was relatively low in the cancer-surrounding normal tissues (“N1/N2/N3/N4”) (Fig. 1E). We also combined the blotting data of all twenty sets of tissues’ results and found that REG3A protein upregulation in breast cancer tissues was significant (*P* < 0.05 versus “N” tissues) (Fig. 1F). The representative tissue immunohistochemistry (IHC) images further supported REG3A protein upregulation in breast cancer tissues (“T1/T2/T3”) of three patients (Fig. 1G). Its expression is again low in cancer-surrounding normal tissues (“N1/N2/N3”) (Fig. 1G).

REG3A expression in various human breast cancer cells was studied next. As demonstrated, the mRNA and protein expression of REG3A was upregulated in primary breast cancer cells (“pBC-1” and “pBC-2”, derived from two different TNBC patients) and in immortalized cell lines (MCF-7 and MDA-231) (Fig. 1H and I). Its expression was relatively low in the primary mammary epithelial cells (pMEC) and MCF-10 A cells (Fig. 1H and I). These results supported REG3A upregulation in breast cancer tissues and cells.

## shRNA-induced knockdown of REG3A potently inhibits breast cancer cell proliferation and migration

**Fig. 2:**
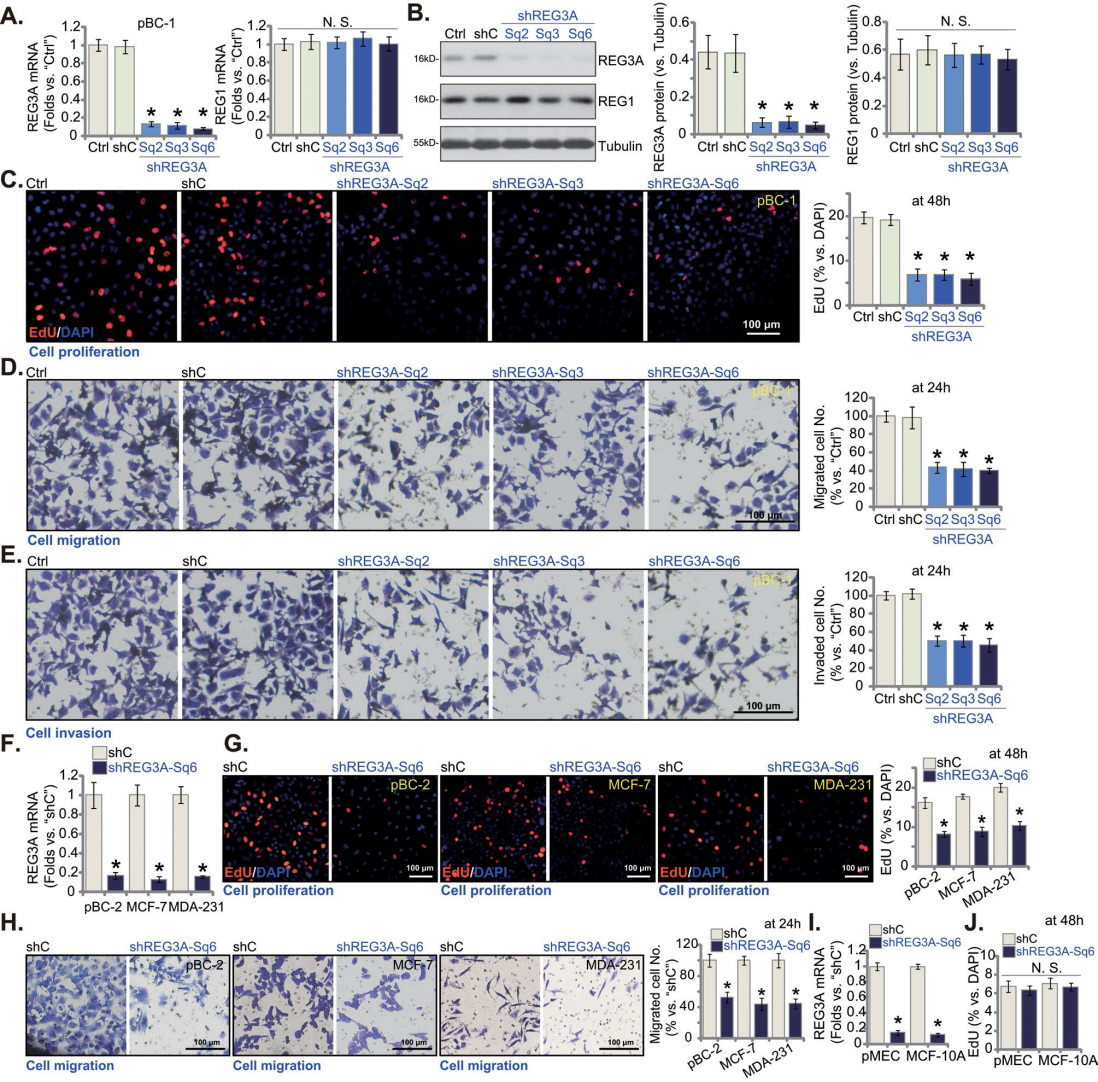
shRNA-induced knockdown of REG3A potently inhibits breast cancer cell proliferation and migration. The mRNA and protein expression of REG3A and REG1 in the stable pBC-1 primary breast cancer cells with the applied REG3A shRNA (“shREG3A-Sq2/3/6”, with non-overlapping sequences), the scramble control shRNA (“shC”), or in the parental control cells (“Ctrl”) was shown (**A** and **B**). Cells were further cultivated for indicated hours, cell proliferation (by measuring nuclear EdU incorporation, **C**), cell migration (**D**) and invasion (**E**) were tested. The pBC-2 primary cancer cells, MCF-7 and MDA-231 lines, the primary mammary epithelial cells (pMEC) or established MCF-10A epithelial cells with shC and shREG3A-Sq6 were formed, and *REG3A* mRNA expression tested (**F** and **I**). Cells were further cultivated for designated hours, cell proliferation (**G** and **J**) and in vitro cell migration (**H**) were examined similarly. Values were mean ± standard deviation (SD, *n* = 5). *P < 0.05 versus “shC” group. “N. S.” stands for non-statistical difference (*P* > 0.05). Experiments were repeated five times and similar results were obtained each time. Scale bar = 100 μm

To test the possible functional role of REG3A in breast cancer cells, we utilized shRNA strategy to genetically silence *REG3A*. Three shRNAs led to substantial *REG3A* mRNA silencing, including shREG3A-Sq2, shREG3A-Sq3, and shREG3A-Sq6 (Fig. 2A) in pBC-1 TNBC cells. REG3A protein expression in pBC-1 cells was downregulated as well by the three shRNAs (Fig. 2B). REG3A shRNAs, however, failed to alter the expression of *REG1* mRNA (Fig. 2A) and proteins (Fig. 2B).

The functional consequences of genetic silencing of REG3A were examined in breast cancer cells. As shown, REG3A silencing by the shRNAs largely inhibited pBC-1 cell proliferation and substantially decreased nuclear EdU incorporation (Fig. 2C). “Transwell” assay results showed that shREG3A-Sq2/3/6 treatment significantly reduced pBC-1 cell in vitro migration (Fig. 2D) and invasion (Fig. 2E). The lentiviral scramble control shRNA (“shC”), as expected, failed to alter REG3A/REG1 expression (Fig. 2A and B) and pBC-1 cell functions (Fig. 2C-E).

Among the tested shRNAs, shREG3A-Sq6 demonstrated superior activity in silencing REG3A. Thus, shREG3A-Sq6-expressing lentivirus was added to other primary TBNC cells, pBC-2, and also to the immortalized cell lines (MCF-7 and MDA-231). It again resulted in substantial *REG3A* mRNA downregulation after stable cell selection (Fig. 2F). Similar to the results in pBC-1 cells, shREG3A-Sq6-induced knockdown of *REG3A* largely inhibited proliferation (EdU incorporation in nuclei, Fig. 2G) and migration (Fig. 2H) in the primary and established breast cancer cells. Whereas in primary mammary epithelial cells (“pMEC”) and MCF-10 A established cells, *REG3A* silencing by shREG3A-Sq6 (Fig. 2I) failed to inhibit nuclear EdU incorporation, the indicator of cell proliferation (Fig. 2J). Thus REG3A silencing potently inhibited breast cancer cell proliferation and migration.

### shRNA-induced silencing of REG3A provokes apoptosis in breast cancer cells

**Fig. 3:**
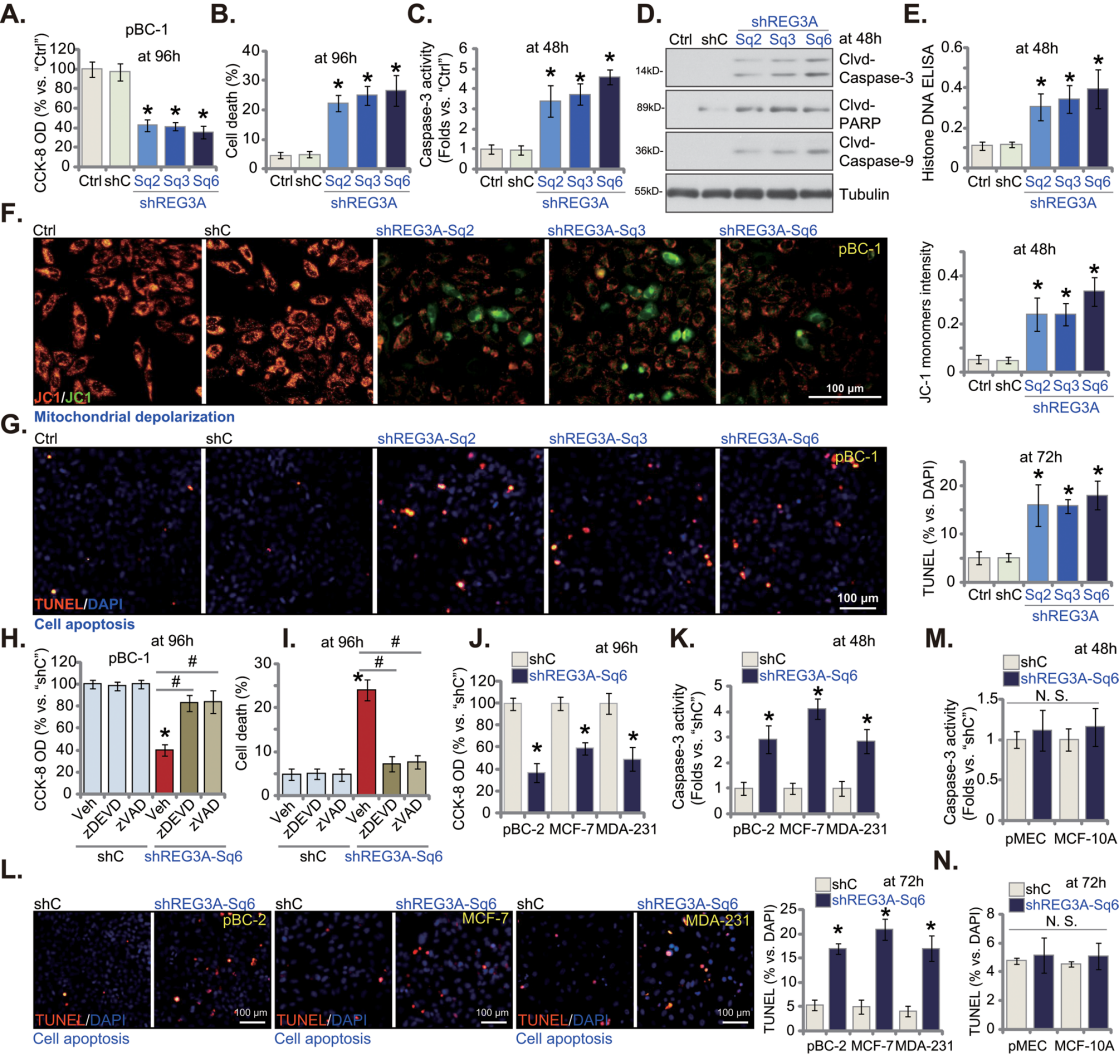
shRNA-induced silencing of REG3A provokes apoptosis in breast cancer cells. The pBC-1 primary breast cancer cells with the applied REG3A shRNA (“shREG3A-Sq2/3/6”, with non-overlapping sequences), the scramble control shRNA (“shC”), as well as the parental control cells (“Ctrl”) were cultivated for indicated time periods, cell viability and death were tested by CCK-8 (**A**) and Trypan blue staining (**B**) assays, respectively; Caspase-PARP activation was examined as well (**C** and **D**); Levels of histone-bound DNA were measured via an ELISA assay (**E**), with mitochondrial depolarization tested by JC-1 fluorescence staining assay (**F**); Cell apoptosis was examined by nuclear TUNEL staining and TUNEL-positive nuclei percentage (% versus DAPI) was calculated (**G**). The pBC-1 primary breast cancer cells with shREG3A-Sq6 or shC were treated with z-DEVD-fmk (“zDEVD”, 30 µM), z-VAD-fmk (“zVAD”, 30 µM) or vehicle control (0.15% DMSO), cells were further cultivated for additional 96 h, cell viability (CCK-8 OD, **H**) and death (Trypan blue staining assays, **I**) were tested. The pBC-2 primary cancer cells, MCF-7 and MDA-231 established cancer cell lines, the primary mammary epithelial cells (pMEC) or established MCF-10A epithelial cells, with shC and shREG3A-Sq6, were cultivated for designated hours, cell viability (CCK-8 OD, **J**), the caspase-3 activity (**K** and **M**) and cell apoptosis (by measuring TUNEL-positive nuclei percentage, **L** and **N**) were tested. Values were mean ± standard deviation (SD, *n* = 5). *P < 0.05 versus “shC” group. ^***#***^*P* < 0.05 (**H** and **I**). “N. S.” stands for non-statistical difference (*P* > 0.05). Experiments were repeated five times and similar results were obtained each time. Scale bar = 100 μm

The results above demonstrated that genetic silencing of REG3A led to growth arrest. Whether cell apoptosis occurred was studied next. In pBC-1 TNBC cells, shREG3A-Sq2/3/6-induced silencing of REG3A (see Fig. 2) led to a significant viability (CCK-8 OD) reduction (Fig. 3A). Significant cell death, as evidenced by increased Trypan blue staining, was also detected in REG3A-silenced pBC-1 TNBC cells (Fig. 3B). In addition, REG3A shRNAs augmented Caspase-3 activity (Fig. 3C) and induced cleavage and activation of key apoptosis implementing proteins, including caspase-3, caspase-9, and poly(ADP-ribose) polymerase 1 (PARP) (Fig. 3D). Furthermore, the levels of Histone-bound DNA were increased in REG3A-silenced pBC-1 cells (Fig. 3E).

Loss of mitochondrial membrane potential (MMP) is a characteristic marker of mitochondrial apoptosis cascade activation [29]. Here, shRNA-induced silencing of REG3A led to mitochondrial depolarization in pBC-1 cells, which was evidenced by the JC-1 transition from red fluorescence aggregates to green fluorescent monomers (Fig. 3F). Additional experimental results showed that apoptosis was induced by REG3A shRNAs and TUNEL-positively stained nuclei were increased (Fig. 3G). The control shC treatment, as expected, failed to provoke caspase-apoptosis activation in pBC-1 cells (Fig. 3A-G). Importantly, apoptosis activation should be the primary mechanism of REG3A shRNA-induced cytotoxicity in breast cancer cells. The caspase-3 specific inhibitor z-DEVD-fmk and the pan-caspase inhibitor z-VAD-fmk each significantly inhibited REG3A silencing (by shREG3A-Sq6)-induced viability reduction (Fig. 3H) and cell death (Fig. 3I) in pBC-1 TNBC cells.

In pBC-2 TNBC cells and immortalized cell lines (MCF-7 and MDA-231), genetic silencing of REG3A by shREG3A-Sq6 (see Fig. 2) led to dramatic viability reduction (Fig. 3J), caspase-3 activation (Fig. 3K), and apoptosis (Fig. 3L); the latter was again evidenced by increased nuclear TUNEL staining (Fig. 3L). Whereas in primary pMEC and established MCF-10 A epithelial cells, silencing of REG3A by shREG3A-Sq6 (see Fig. 2) failed to provoke caspase-3 and apoptosis activation (Fig. 3M and N).

### REG3A knockout produces significant anti-breast cancer cell activity

**Fig. 4:**
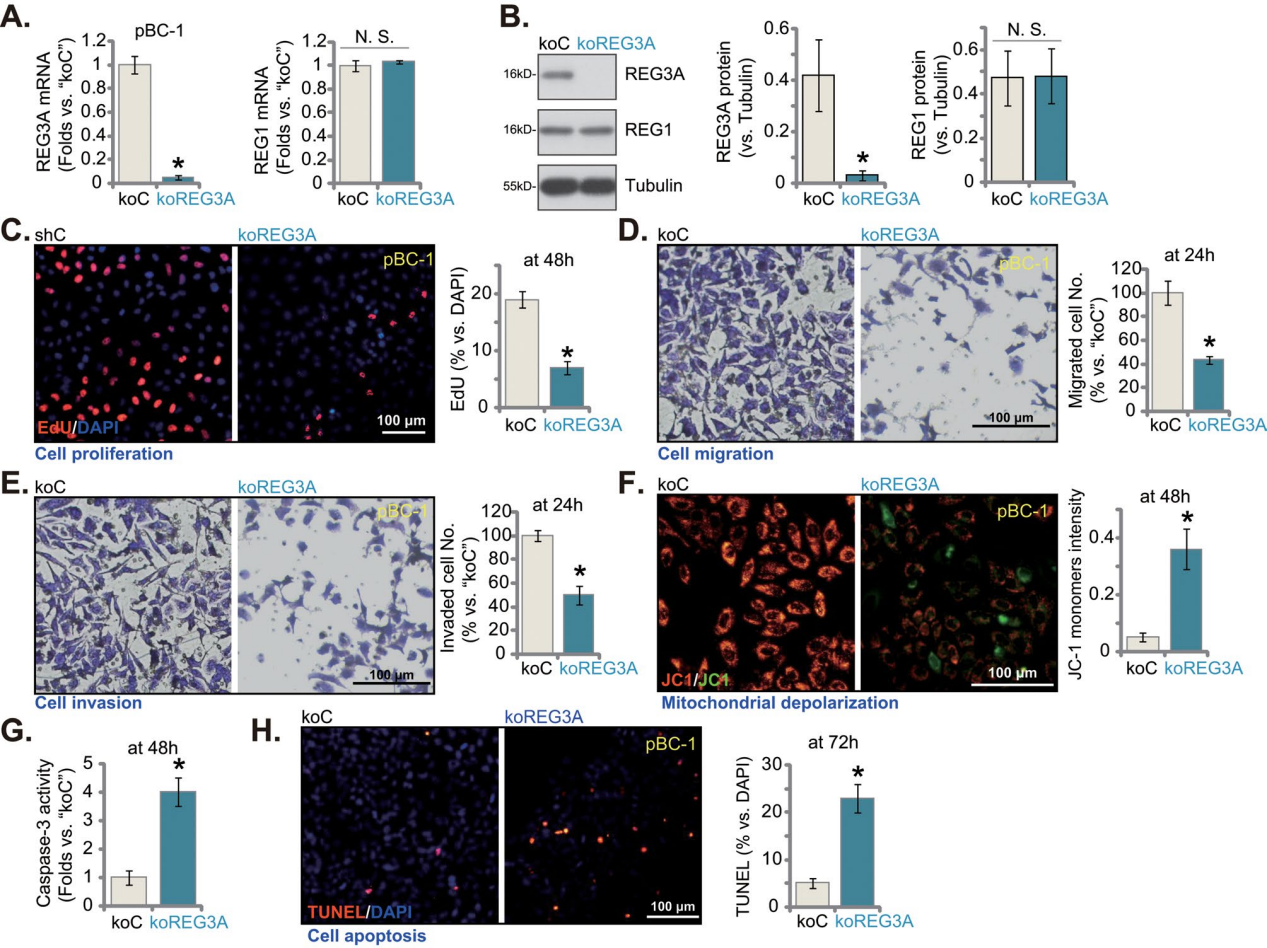
REG3A knockout produces significant anti-breast cancer cell activity. The single stable pBC-1 cells, with the lentiviral CRISPR/Cas9-REG3A-KO construct (“koREG3A”) or the CRISPR/Cas9-control construct (“koC”), were established and expression of listed mRNAs and proteins was shown (**A** and **B**). Cells were further cultivated for indicated hours, cell proliferation (by measuring EdU incorporation, **C**), cell migration (**D**) and invasion (**E**) were tested; Mitochondrial depolarization (JC-1 green monomers formation, **F**); Caspase-3 activity (**G**) and cell apoptosis (TUNEL-positive nuclei percentage, **H**) were measured as well. Values were mean ± standard deviation (SD, *n* = 5). *P < 0.05 versus “koC” group. “N. S.” stands for non-statistical difference (*P* > 0.05). Experiments were repeated five times and similar results were obtained each time. Scale bar = 100 μm

To completely deplete *REG3A*, the CRISPR/Cas9 knockout (KO) strategy was employed. As shown, mRNA and protein expression of REG3A were depleted in koREG3A pBC-1 TNBC cells (Fig. 4A and B). Contrarily, *REG1* mRNA and protein expression was intact (Fig. 4A and B). Testing pBC-1 cell functions demonstrated that CRISPR/Cas9-induced REG3A KO substantially inhibited cell proliferation and decreased the ratio of EdU-positive nuclei (Fig. 4C). Moreover, pBC-1 cell in vitro migration (Fig. 4D) and invasion (Fig. 4E) were significantly suppressed with REG3A KO. Further studies showed that REG3A KO caused the accumulation of JC-1 green fluorescence monomers in pBC-1 cells, supporting mitochondrial depolarization (Fig. 4F). Moreover, the caspase-3 activity (Fig. 4G) and the TUNEL positively-stained nuclei percentage (Fig. 4H) were both significantly increased in koREG3A pBC-1 cells, supporting apoptosis activation. Together, these results showed that REG3A KO led to significant anti-cancer cell activity in primary breast cancer cells.

### REG3A overexpression results in cancer-promoting activity in breast cancer cells

**Fig. 5:**
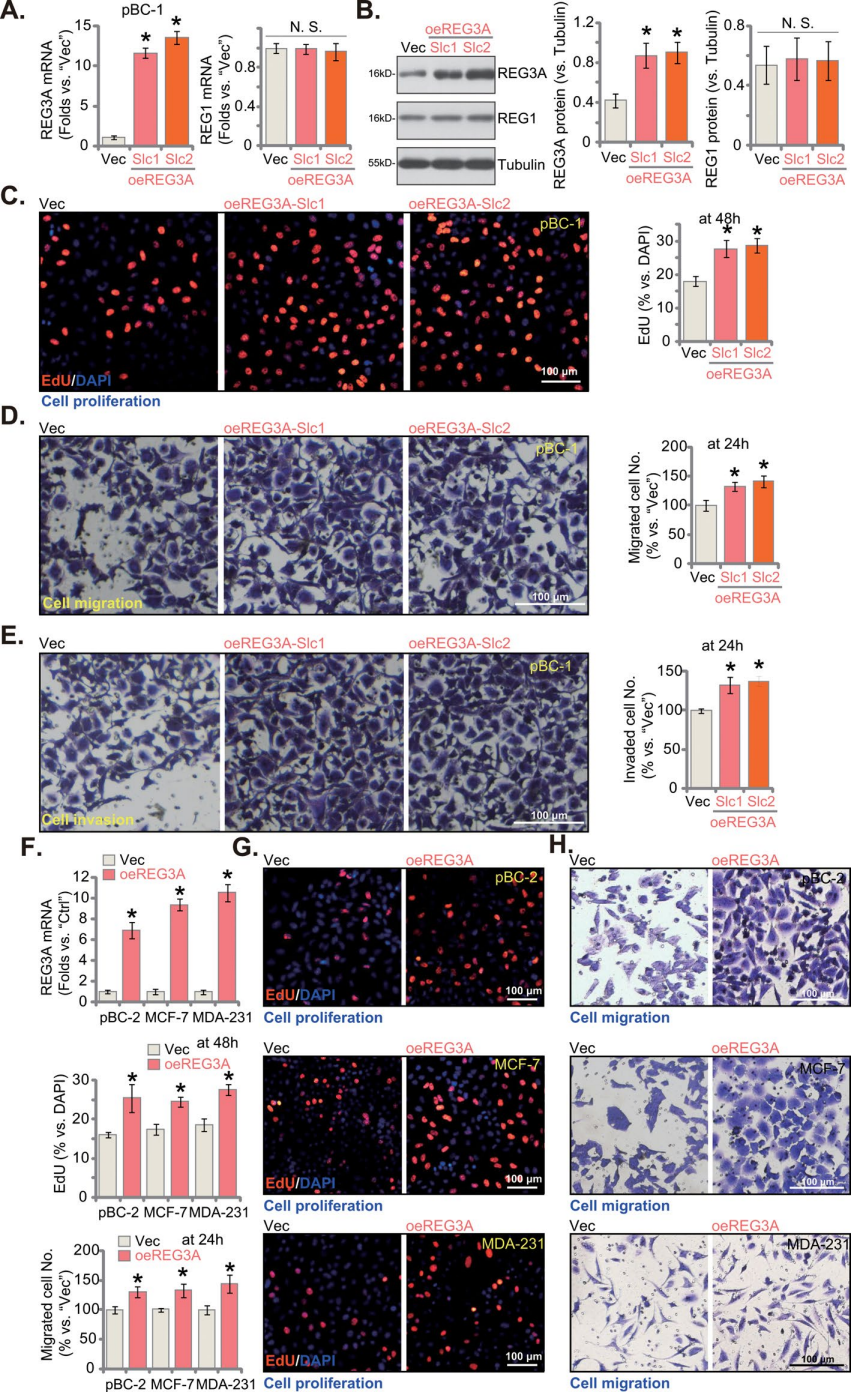
REG3A overexpression results in cancer-promoting activity in breast cancer cells. The pBC-1 primary breast cancer cells expressing the REG3A-overexpressing construct (oeREG3A-Slc1 and oeREG3A-Slc2, representing two stable selections) or the empty vector (“Vec”) were established, and expression of listed mRNAs and proteins was shown (**A** and **B**). Cells were further cultivated for indicated hours, cell proliferation (by measuring EdU incorporation, **C**), cell migration (**D**) and invasion (**E**) were tested; The pBC-2 primary breast cancer cells or established lines (MCF-7 and MDA-231) with the lentiviral REG3A-overexpressing construct (“oeREG3A”) or the empty vector (“Vec”) were established, and expression of *REG3A* mRNA was shown (**F**); Cells were further cultivated for designated time periods, cell proliferation (**G**) and migration (**H**) were tested similarly. Values were mean ± standard deviation (SD, *n* = 5). *P < 0.05 versus “Vec” group. “N. S.” stands for non-statistical difference (*P* > 0.05). Experiments were repeated five times and similar results were obtained each time. Scale bar = 100 μm

Since REG3A silencing/KO resulted in potent anti-breast cancer cell activity, we hypothesized that ectopic overexpression of REG3A could then exert opposite functions. As compared with the control cells with the empty vector (“Vec”), *REG3A* mRNA expression increased over ten fold in oeREG3A-Slc1/-Slc2 pBC-1 TNBC cells (Fig. 5A). REG3A protein upregulation was detected as well (Fig. 5B). The mRNA and protein levels of REG1 were again unchanged (Fig. 5A and B). Cellular functional studies revealed that ectopic overexpression of REG3A strengthened pBC-1 cell proliferation and increased the EdU-incorporated nuclei ratio (Fig. 5C). Moreover, in vitro cell migration (Fig. 5D) and invasion (Fig. 5E) were augmented in oeREG3A-Slc1/-Slc2 pBC-1 cells.

The REG3A-overexpressing lentivirus was also added to pBC-2 TNBC cells and immortalized lines (MCF-7 and MDA-231). Following stable cell selection by puromycin, stable cells (“oeREG3A”) were formed. These cells showed significantly upregulated *REG3A* mRNA expression (Fig. 5F). Similar to the results in pBC-1 cells, oeREG3A also augmented cell proliferation (Fig. 5G) and accelerated cell migration (Fig. 5H) in primary and immortalized breast cancer cells. Together, we showed that REG3A overexpression resulted in cancer-promoting activity in breast cancer cells.

### REG3A is vital for Akt-mTOR activation in breast cancer cells

**Fig. 6:**
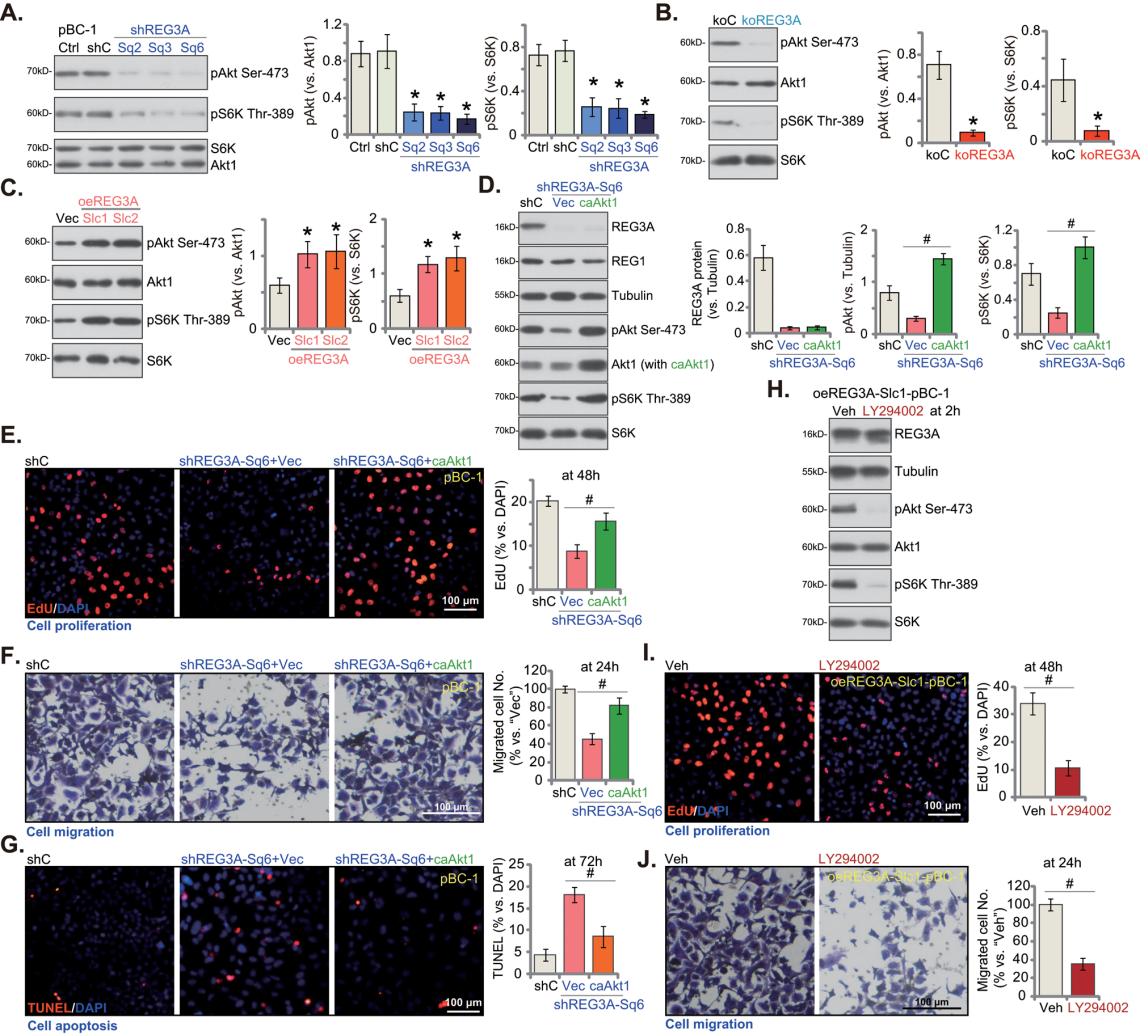
REG3A is vital for Akt-mTOR activation in breast cancer cells. The pBC-1 primary breast cancer cells, with the applied REG3A shRNA (“shREG3A-Sq2/3/6”, with non-overlapping sequences), the scramble control shRNA (“shC”), the lentiviral CRISPR/Cas9-REG3A-KO construct (“koREG3A”), the CRISPR/Cas9-control construct (“koC”), REG3A-overexpressing construct (oeREG3A-Slc1 and oeREG3A-Slc2, two stable selections) or the empty vector (“Vec”), were cultured and expression of listed proteins was shown (**A**-**C**). The pBC-1 primary breast cancer cells with shREG3A-Sq6 were further stably transduced with the viral constitutively-active mutant S473D Akt1 (caAkt1) construct or the empty vector (“Vec”), expression of listed proteins was shown (**D**). Cells were further cultivated for designated hours, cell proliferation, migration and apoptosis were examined respectively through nuclear EdU staining (**E**), “Transwell” (**F**) and nuclear TUNEL staining (**G**) assays. The oeREG3A-Slc1 pBC-1 primary cells were treated with LY294002 or vehicle control (“Veh”, 0.15% DMSO) for designated hours, expression of listed proteins was shown (**H**); Cell proliferation (**I**) and migration (**J**) were tested using the same methods. Values were mean ± standard deviation (SD, *n* = 5). *P < 0.05 versus “shC”/“koC”/“Vec” group. ^***#***^*P* < 0.05. Experiments were repeated five times and similar results were obtained each time. Scale bar = 100 μm

Due to various genetic mutations, Akt-mTOR cascade is often hyper-activated in TNBC, serving as a key protein for cancer progression [30–36]. We thus analyzed the potential effect of REG3A on Akt-mTOR cascade activation. pBC-1 TNBC cells were *PTEN*-null cells and showed high basal phosphorylation of Akt (at Ser-473) and S6K (at Thr-389) (Fig. 6A). Importantly, REG3A silencing by different shRNAs (shREG3A-Sq2/3/6, see Figs. 2 and 3) largely inhibited Akt and S6K phosphorylation in pBC-1 cells (Fig. 6A). Total Akt1 and S6K protein levels were unchanged with REG3A knockdown (Fig. 6A). Moreover, in pBC-1 cells, CRISPR/Cas9-induced KO of REG3A (see Fig. 4) also remarkably decreased Akt1 and S6K phosphorylation (Fig. 6B), without affecting total Akt1 and S6K expression (Fig. 6B). Contrarily, in REG3A-overexpressed pBC-1 cells, oeREG3A-Slc1/2 (see Fig. 5), Akt-S6K phosphorylation was significantly augmented (Fig. 6C). The results support that REG3A is important for Akt-mTOR activation in breast cancer cells.

To understand the role of Akt-mTOR activation in REG3A-mediated breast cancer cell growth, a viral constitutively-active mutant S473D Akt1 (“caAkt1”) construct was stably transduced to shREG3A-Sq6-expressing pBC-1 cells. As shown, caAkt1 restored Akt and S6K phosphorylation in REG3A-silenced pBC-1 cells (Fig. 6D). Expectably, caAkt1 did not affect REG3A protein expression (Fig. 6D). The functional studies showed that caAkt1 largely ameliorated REG3A silencing-induced proliferation arrest (EdU assays, Fig. 6E), migration inhibition (Fig. 6F), and apoptosis (Fig. 6G) in pBC-1 cells. Thus, mediating Akt-mTOR activation could be a primary mechanism of REG3A-driven breast cancer cell growth.

To further support the role of Akt-mTOR cascade in REG3A-mediated actions, the pan PI3K-Akt-mTOR inhibitor LY294002 [37] was utilized. In oeREG3A-Slc1 pBC-1 primary cells (see Fig. 5), treatment with LY294002 (at 1 µM) blocked Akt-S6K1 phosphorylation (Fig. 6H). Once again, REG3A protein expression as well as total Akt1 and S6K1 expression were unaffected by LY294002 (Fig. 6H). After LY294002 treatment, pBC-1 cell proliferation (by calculating EdU-incorporated nuclei percentage, Fig. 6I) and migration (Fig. 6J) were substantially decreased.

### REG3A is important for maintaining the integrity of mTOR complexes

**Fig. 7:**
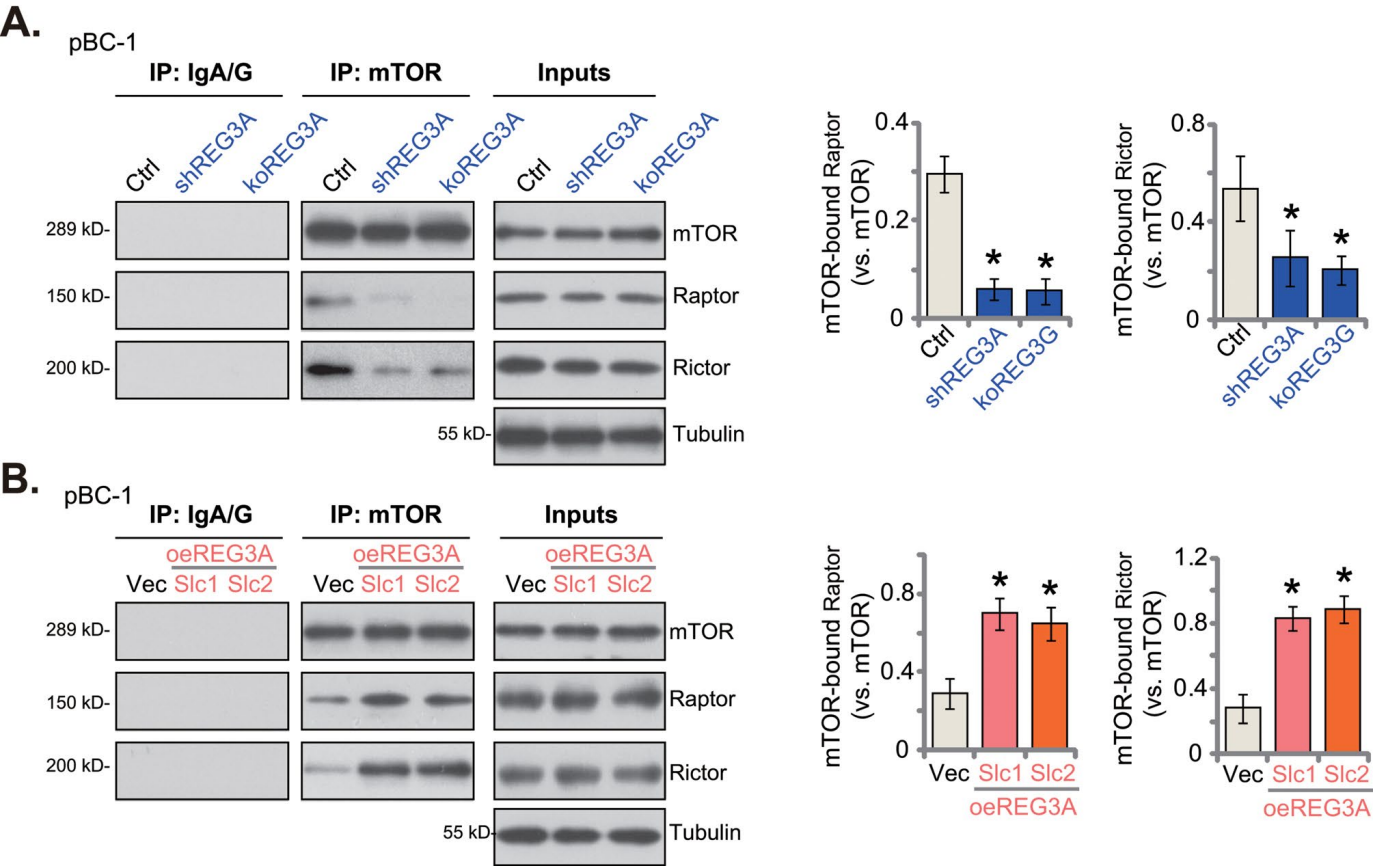
REG3A is important for maintaining the integrity of mTOR complexes. The pBC-1 primary breast cancer cells, with shREG3A-Sq6 (“shREG3A”), the lentiviral CRISPR/Cas9-REG3A-KO construct (“koREG3A”), REG3A-overexpressing construct (oeREG3A-Slc1 and oeREG3A-Slc2, two stable selections) or the empty vector (“Vec”), were cultured, mTOR-Raptor-Rictor associations were examined via co-immunoprecipitation (“IP”) assays (**A** and **B**), with expression of the listed proteins tested as “Inputs” (**A** and **B**). “Ctrl” stands for the parental control cells (**A**). Values were mean ± standard deviation (SD, *n* = 5). *P < 0.05 versus “Ctrl”/“Vec” group. Experiments were repeated five times and similar results were obtained each time

Next, we examined the possible underlying mechanism of REG3A-promoted Akt-mTOR activation. The mTOR complex 1 (mTORC1), a multiple-protein complex including mTOR, Raptor, and mLST8, phosphorylates S6K1 and 4EBP1 [38–43]. The other mTOR protein complex, mTORC2, is assembled with mTOR, Rictor, Sin1, mLST8, and several others, and phosphorylates Akt (Ser-473) and other AGC kinases [38–43]. The co-immunoprecipitation assay results, Fig. 7A, demonstrated that REG3A silencing (by “shREG3A-Sq6”, see Figs. 2 and 3) or knockout ( “koREG3A”, see Fig. 4) potently inhibited mTOR-Raptor association (mTORC1 assemble) and mTOR-Rictor association (mTORC2 assemble) in pBC-1 TNBC cells. However, there was no change in the expression levels of the mTOR, Rictor, and Raptor proteins of the mTOR complex (Fig. 7A, “Input”). Contrarily, in REG3A-overexpresed pBC-1 cells (“oeREG3A-Slc1” and “oeREG3A-Slc2”, see Fig. 5), mTOR-Raptor association (Fig. 7B) and mTOR-Rictor association (Fig. 7B) were both augmented. Once again, mTOR, Rictor, and Raptor expression was unchanged with REG3A overexpression (Fig. 7B, “Input”). Therefore, REG3A could be important for maintaining the integrity of both mTORC1 and mTORC2, thereby promoting Akt-mTOR activation in breast cancer cells.

### ZNF680 is a potential transcription factor of REG3A in breast cancer cells

**Fig. 8:**
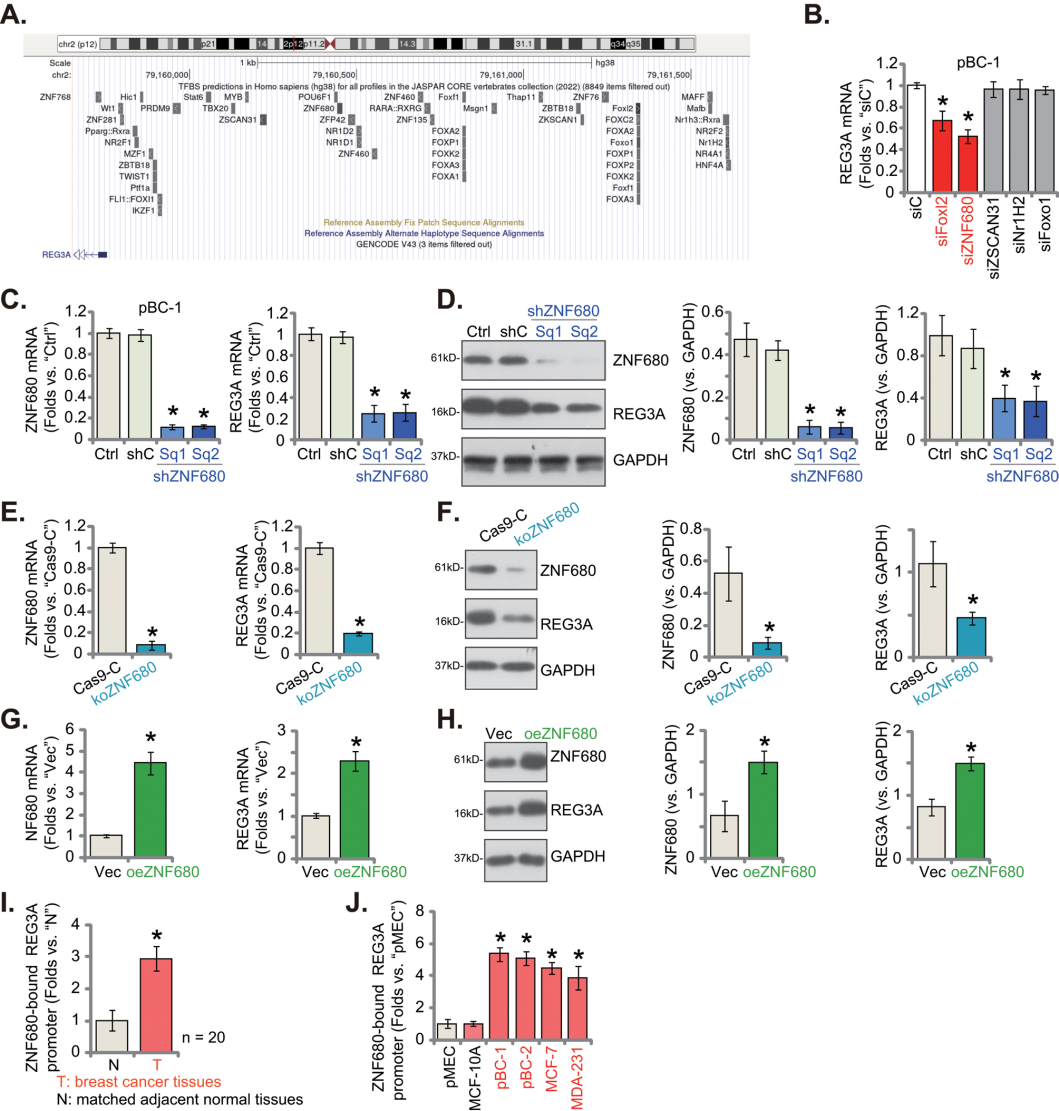
ZNF680 is a potential transcription factor of REG3A in breast cancer cells. The JASPAR database predicted potential transcription factors for REG3A (**A**). Following transfection of pBC-1 cells with the specified siRNAs targeting various transcription factors or a scramble non-sense siRNA (siC) for 48 h, the expression of *REG3A* mRNA was assessed (**B**). pBC-1 cells were then subjected to lentiviral ZNF680 shRNA (shZNF680-Sq1 or shZNF680-Sq2) and scramble control shRNA (“shC”) treatments (**C** and **D**), lentiviral CRISPR/Cas9-ZNF680-KO construct (“koZNF680”) and CRISPR/Cas9 control construct (“Cas9-C”) treatments (**E** and **F**), as well as lentiviral ZNF680-expressing construct (oeZNF680) or an empty vector (“Vec”) treatments (**G** and **H**), resulting in the establishment of stable cells. The expression of the listed mRNAs and proteins was then evaluated (**C**-**H**). Chromatin Immunoprecipitation (ChIP) assay results demonstrated the relative levels of ZNF680-bound *REG3A* promoter in specified breast tumor tissues (“T”) and matched adjacent normal tissues (“N”) (**I**) as well as in listed breast cancer cells and pMEC/MCF-10 A cells (**J**). “Ctrl” stands for the parental control cells. Values were mean ± standard deviation (SD). * *P* < 0.05 versus “siC” (**B**). * *P* < 0.05 versus “shC”/“Cas9-C”/“Vec” cells (**C**-**H**). * *P* < 0.05 versus “N” tissues or pMEC cells (**I** and **J**). Experiments were repeated five times and similar results were obtained each time

Given the observed elevation in both mRNA and protein expression of REG3A in breast cancer tissues and cells, our hypothesis centered on the possibility of a transcriptional mechanism contributing to the upregulation. As limited studies have explored the transcription factors associated with REG3A, we conducted a search using the JASPAR transcription factor database. Five transcription factors exhibiting the highest potential binding affinity to REG3A were identified: Foxl2, ZNF680, ZSCAN31, Nr1H2, and Foxo1 (Fig. 8A). To explore their impact on *REG3A* mRNA expression, we designed siRNAs targeting each of these transcription factors and individually transfected them into pBC-1 TNBC cells. Only Foxl2 siRNA and ZNF680 siRNA led to a noteworthy silencing of REG3A in pBC-1 cells (Fig. 8B). Silencing other transcription factors had no significant effect (Fig. 8B). Notably, ZNF680 siRNA exhibited a greater potency than Foxl2 siRNA in downregulating REG3A (Fig. 8B).

Subsequently, pBC-1 cells were treated with the lentivirus carrying ZNF680 shRNA (shZNF680-Sq1 and shZNF680-Sq2, utilizing different sequences), and stable cells were generated. In contrast to control pBC-1 cells treated with scramble control shRNA (“shC”), pBC-1 cells with shZNF680 exhibited a marked reduction in ZNF680 mRNA (Fig. 8C) and protein levels (Fig. 8D), accompanied by a significant downregulation of REG3A mRNA (Fig. 8C) and protein (Fig. 8D) expression. As an alternative strategy, pBC-1 cells expressing Cas-9 were stably transduced with a lentiviral CRISPR/Cas9-ZNF680-KO construct, resulting in the development of ZNF680 knockout cells (“koZNF680”). In these koZNF680 pBC-1 cells, both the mRNA (Fig. 8E) and protein (Fig. 8F) expression of ZNF680 was depleted, and REG3A expression was substantially reduced.

To further substantiate our hypothesis, pBC-1 cells were exposed to the lentivirus carrying the ZNF680-expressing construct. Stable cells, “oeZNF680,” were established through puromycin-based selection. These cells exhibited pronounced upregulation of ZNF680 mRNA (Fig. 8G) and protein levels (Fig. 8H). Notably, the overexpression of ZNF680 resulted in a concurrent upregulation of *REG3A* mRNA (Fig. 8G) and protein expression (Fig. 8H). Importantly, ChIP assay results revealed a significant increase in the binding between ZNF680 protein and the putative REG3A promoter region (as identified in the JASPAR database) in breast cancer tissues from local patients (Fig. 8I). Furthermore, this enhanced binding was consistently observed in various primary and immortalized breast cells (pBC-1, pBC-2, MCF-7, and MDA-231) (Fig. 8J). In contrast, the ZNF680-REG3A promoter binding affinity was relatively low in normal breast tissues (“N”) (Fig. 8I) and in pMEC/MCF-10 A cells (Fig. 8J). These findings indicate that ZNF680 is an important transcription factor for REG3A, and the enhanced binding between ZNF680 and the *REG3A* promoter may represent a key mechanism contributing to the upregulation of REG3A in breast cancer.

### REG3A silencing impedes breast cancer xenograft growth in nude mice

**Fig. 9:**
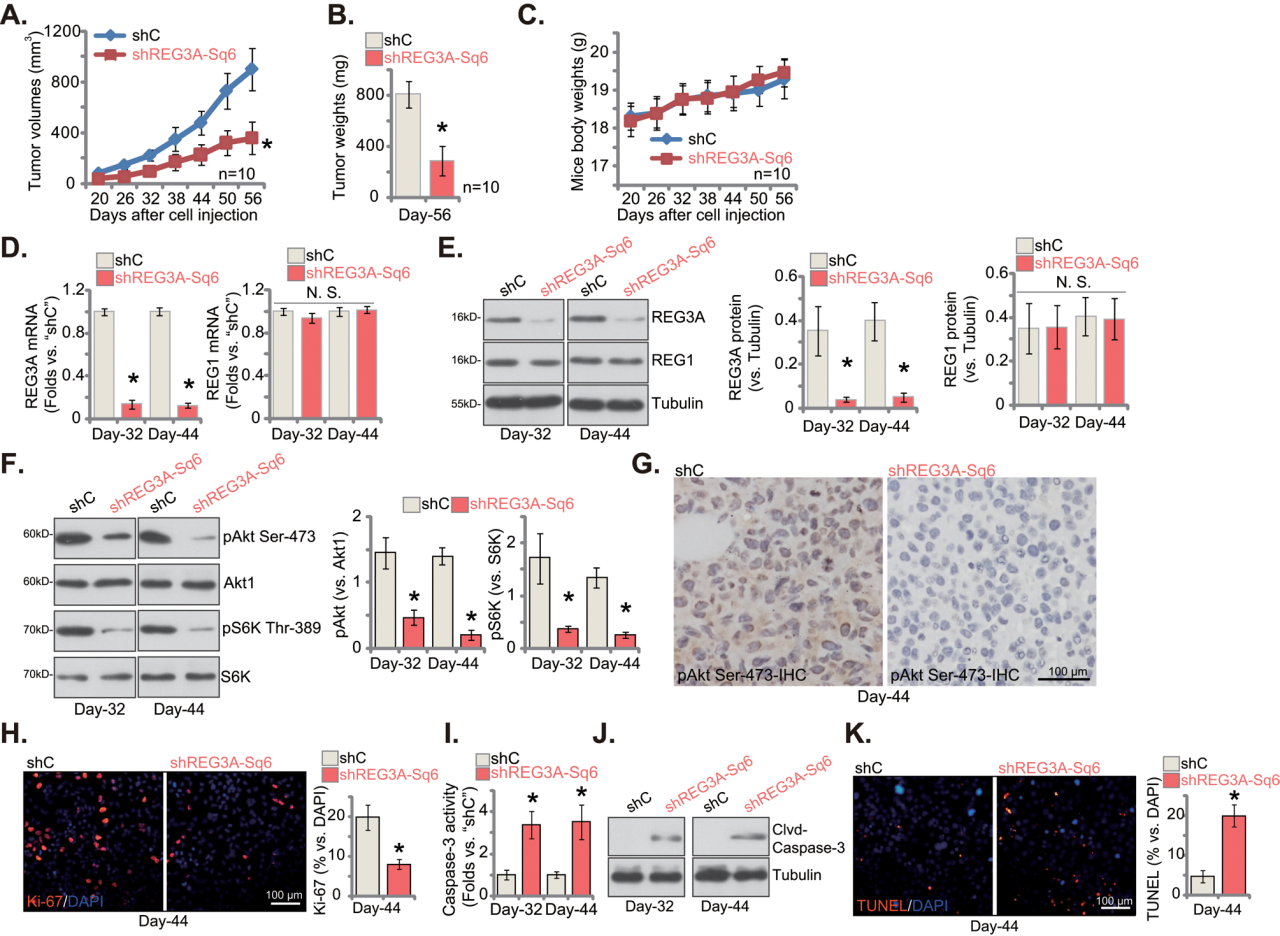
REG3A silencing impedes breast cancer xenograft growth in nude mice. The pBC-1 primary breast cancer cells, with REG3A shRNA (“shREG3A-Sq6”) or scramble control shRNA (“shC”), were subcutaneously (*s.c.*) injected to the flanks of the nude mice to form xenografts. The pBC-1 xenograft volumes (**A**) and animal body weights (**C**) were recorded starting at Day-22 (22 days after cell injection) and every six days after. At Day-56, all pBC-1 xenografts were isolated and weighted (**B**); At Day-32 and Day-44, one pBC-1 xenograft in each group was isolated and total four xenografts were obtained. Expression of listed mRNAs and proteins in fresh xenograft tissue lysates was tested (**D**, **E**, **F** and **J**); The relative caspase-3 activity was measured as well (**I**). Alternatively, pBC-1 xenograft slides were subject to immunohistochemistry (IHC) staining of pAkt (Ser-473) (**G**). Moreover, nuclear Ki-67/DAPI fluorescence staining (**H**) and nuclear TUNEL/DAPI fluorescence staining (**K**) were carried out in the described pBC-1 xenograft slides. Values were mean ± standard deviation (SD). In **A**-**C**, ten mice per group (*n* = 10). For **D**-**K**, five random tissue pieces in each xenograft were tested. *P < 0.05 versus “shC” group. “N. S.” indicated no statistical difference (*P* > 0.05). Scale bar = 100 μm

At last, the potential role of REG3A on the growth of breast cancer cells in vivo was explored. The pBC-1 TNBC cells (at five million cells per mouse), with REG3A shRNA (“shREG3A-Sq6”) or control shRNA (“shC”), were injected into the nude mice flanks. The xenograft recordings started 20 days after cell injection, every six days after. The xenograft growth curve results in Fig. 9A demonstrated that the growth of shREG3A-Sq6-expressing pBC-1 xenografts was remarkably inhibited. The volumes of shREG3A-Sq6 pBC-1 xenografts were significantly lower than those of shC-expressing ones (Fig. 9A). Fifty-six days (“Day-56”) after initial pBC-1 cell injection, all xenografts were isolated and measured. As demonstrated, shREG3A-Sq6-expressing pBC-1 xenografts were significantly lighter and smaller than shC ones (Fig. 9B). There was, however, no significant difference in the animal body weights of the two groups of nude mice (Fig. 9C).

Thirty-two (“Day-32”) and forty-four (“Day-44”) days after initial pBC-1 cell injection, one pBC-1 xenograft per group was isolated, and a tal of four pBC-1 xenografts were obtained. Part of each xenograft was cut into small pieces, and signaling changes were detected. As demonstrated, *REG3A* mRNA and protein expression were substantially decreased in shREG3A-Sq6-expressing pBC-1 xenograft tissues (Fig. 9D and E), while *REG1A* mRNA and protein expression were unchanged (Fig. 9D and E). Activation of Akt-mTOR cascade, or Akt-S6K phosphorylation, was largely inhibited in REG3A-silenced pBC-1 xenograft tissues (Fig. 9F). Part of the xenograft tissues were sectioned into tumor slides, and immunohistochemistry (IHC) results further confirmed Akt (S473 phosphorylation) inhibition after REG3A silencing (Fig. 9G).

The xenograft tissue immunofluorescence images showed that Ki-67-positive nuclei percentage was significantly decreased in pBC-1 xenografts with shREG3A-Sq6, supporting proliferation inhibition in REG3A-silenced xenografts (Fig. 9H). Further studies demonstrated that the Caspase-3 activity was significantly increased in shREG3A-Sq6-expressing pBC-1 xenograft tissues (Fig. 9I), where cleaved Caspase-3 levels were increased (Fig. 9J). Moreover, TUNEL-positive nuclei were increased in shREG3A-Sq6-expressing pBC-1 xenograft slides (Fig. 9K). These results further supported apoptosis activation in REG3A-silenced pBC-1 xenografts. Together, Akt-mTOR inactivation, proliferation inhibition, and apoptosis activation were detected in pBC-1 xenografts after REG3A silencing.

## Discussion

TNBC and other advanced breast cancers continue to stand as the foremost cause of cancer-related morbidity and mortality among women globally [3, 4, 44]. In operable cases, advancements in systemic therapies, including adjuvant and neoadjuvant treatments, have notably enhanced patient outcomes and survival rates, particularly in developed nations [3, 4, 44]. The ongoing development and evaluation of novel molecularly-targeted agents hold promise for specific histological types of breast cancer [3, 4, 44]. However, for patients grappling with therapy-resistant, metastatic, or recurrent breast cancers, the prognosis remains bleak [3, 4, 44]. necessitating the exploration of fresh therapeutic avenues for this debilitating disease [3, 4, 44].

**Fig. 10:**
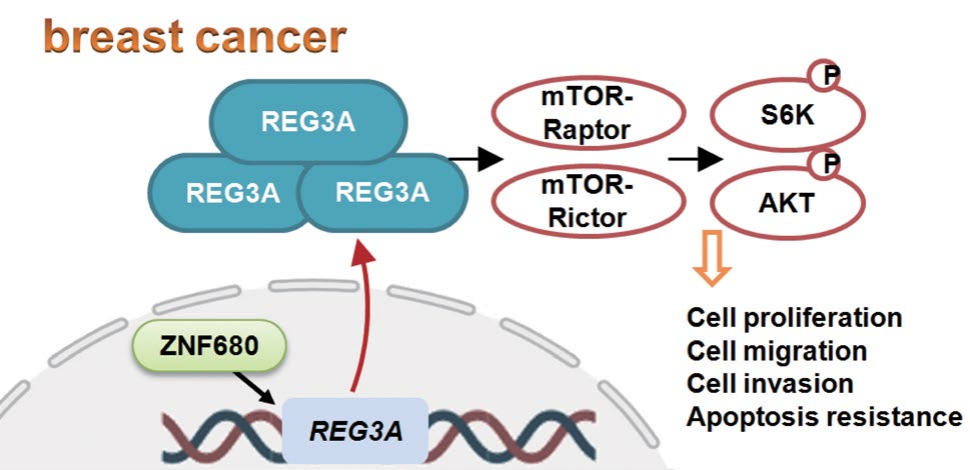
The proposed signaling pathway outlined in this study

Recent studies have underscored REG3A’s potential role in the initiation and advancement of several cancers, primarily exerting pro-proliferation and anti-apoptosis activities [14–17, 45]. Our findings support REG3A as a plausible therapeutic target for TNBC. Analysis from the TCGA database reveals an upsurge in REG3A transcripts within breast cancer tissues. Additionally, both *REG3A* mRNA and protein levels were notably elevated in TNBC tissues from locally treated patients, contrasting sharply with low expression in adjacent normal tissues. In primary TNBC cells, targeted REG3A knockdown (utilizing virus-delivered shRNA) or knockout (via CRISPR sgRNA method) substantially curbed cell proliferation, migration, and invasion. Conversely, enforced overexpression of REG3A accentuated the proliferation and migration of TNBC cells. Notably, in vivo experiments showcased that targeted REG3A silencing via specific shRNA significantly impeded the growth of TNBC cell xenografts in nude mice. These findings underscore the importance of REG3A in fostering breast cancer cell growth both in vitro and in vivo (Fig. 10).

Due to various genetic mutations, including *PIK3CA* mutation and *HER2* overexpression, overactivation of PI3K-Akt-mTOR cascade is important for the progression of TNBC [36] and other breast cancers [33, 35]. It promotes cancer cell proliferation, growth, and migration, as well as glucose metabolism, DNA repair, and apoptosis resistance [33, 35]. Resistance to ER-targeted therapies, either *de novo* or acquired, is persistent in a large proportion of breast cancer patients, which is a key factor in disease progression [34]. PI3K-Akt-mTOR activation plays an important role in ER therapy resistance and thus represents a key therapeutic target [34]. Several PI3K-Akt-mTOR kinase inhibitors are being utilized for the therapy of specific breast cancers [33–36]. In addition, novel inhibitors are being tested and have shown promising therapeutic value for breast cancer patients [33–36].

Lai et al. reported that REG3A activated PI3K-Akt signaling cascade to promote keratinocyte proliferation, and PI3K-Akt inhibitors almost blocked keratinocyte proliferation by REG3A [46]. Ye et al. reported a positive correlation between REG3A overexpression and increased Akt phosphorylation in colorectal cancer cells [15]. A recent study by Jiang and colleagues reported that REG3A enhanced the proliferation of ovarian cancer cells through activating PI3K/Akt signaling pathway [47]. Additionally, REG3A is involved in the activation of the Akt cascade in glioma cells [48]. However, Qiu et al. found that overexpression of REG3A suppressed the PI3K-Akt signaling pathway in gastric cancer cells [19]. The role of REG3A in the activation of mTOR, a critical component of the PI3K-Akt pathway, in cancer cells has not been extensively investigated.

One important finding of this study is that REG3A is important for Akt-mTOR cascade activation in breast cancer cells. Akt-S6K1 phosphorylation was remarkably decreased following REG3A silencing or KO in pBC-1 TNBC cells. It was however augmented after REG3A overexpression. Restoring Akt-mTOR activation by caAkt1 largely ameliorated REG3A knockdown-induced proliferation arrest and apoptosis in pBC-1 cells. Whereas the Akt-mTOR inhibitor, LY294002, inhibited REG3A overexpression-induced pro-cancerous activity in the TNBC cells. In REG3A-silenced TNBC xenograft tissues, Akt-mTOR inhibition was detected. Importantly, REG3A is essential for maintaining the integrity of mTOR complexes, and REG3A depletion disrupted mTOR-Raptor association and mTOR-Rictor association in TBNC cells, whereas the integrity of mTORC1 and mTORC2 was strengthened following REG3A overexpression. Thus, REG3A is important for Akt-mTOR activation in TNBC cells, possibly by maintaining mTOR complex integrity (see the proposed signaling pathway in Fig. 10).

In our investigation, we delved into understanding the potential mechanism behind the increased expression of REG3A, pinpointing ZNF680 as a critical transcription factor for REG3A. In primary TNBC cells, reducing ZNF680 expression through knockdown or knockout methods led to a decrease in REG3A levels, whereas its overexpression augmented REG3A expression at both mRNA and protein levels. We observed enhanced binding between the ZNF680 protein and the REG3A promoter in various breast cancer tissues and primary/immortalized cells. Research on ZNF680’s role in human cancer remains limited, warranting further studies to comprehend its implications for the growth and progression of TNBC cells.

## Conclusion

ZNF680-induced upregulation of REG3A triggers tumorigenesis in breast cancer, potentially by promoting the activation of Akt-mTOR signaling. REG3A emerges as a promising and innovative target for TNBC and possible other advanced breast cancers.

### Electronic supplementary material

Below is the link to the electronic supplementary material.

Supplementary Material 1

## Data Availability

No datasets were generated or analysed during the current study.

## Abbreviations

EdU: 5-ethynyl-2’-deoxyuridine
CCK-8: cell counting kit-8
ChIP: chromatin immunoprecipitation
DMBT1: deleted in malignant brain tumors 1
HCC: hepatocellular carcinoma
IHC: immunohistochemistry
KO: knockout
OD: optical density
IACUC: Institutional Animal Care and Use Committee
qRT-PCR: quantitative real-time PCR (
REG3A: regenerating islet-derived protein 3 A
SD: standard deviation
TUNEL: triple negative breast cancer, terminal deoxynucleotidyl transferase dUTP nick end labeling
TCGA: The Cancer Genome Atlas
