# Supplementary material for: Increased expression of REG3A promotes tumorigenic behavior in triple negative breast cancer cells
Source: Breast Cancer Res. 2024 Jun 5;26:92. doi: 10.1186/s13058-024-01845-2 (PMC11151570; doi:10.1186/s13058-024-01845-2)

Figure S1. The un-cropped blotting images of the study

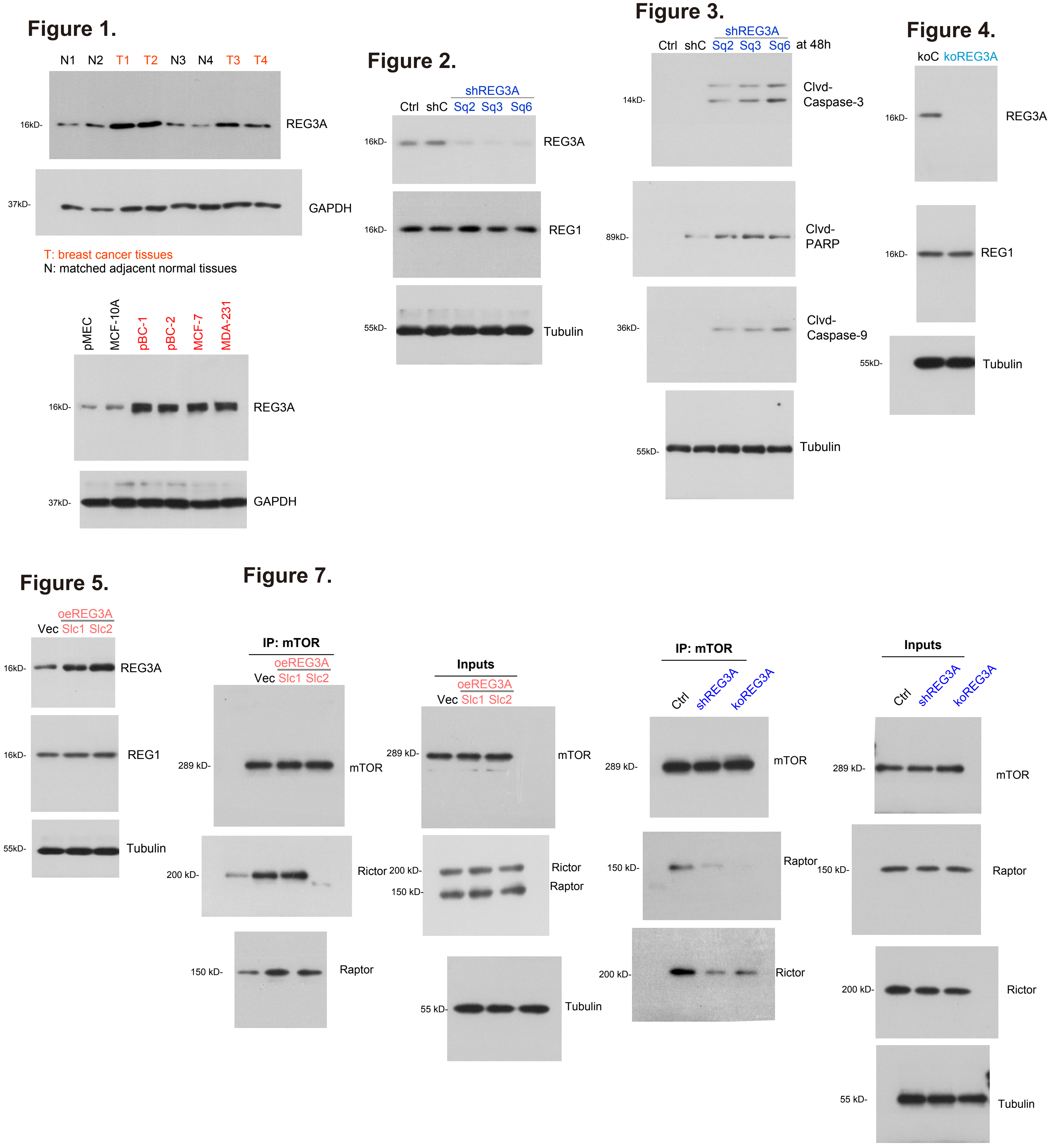

Figure 6.

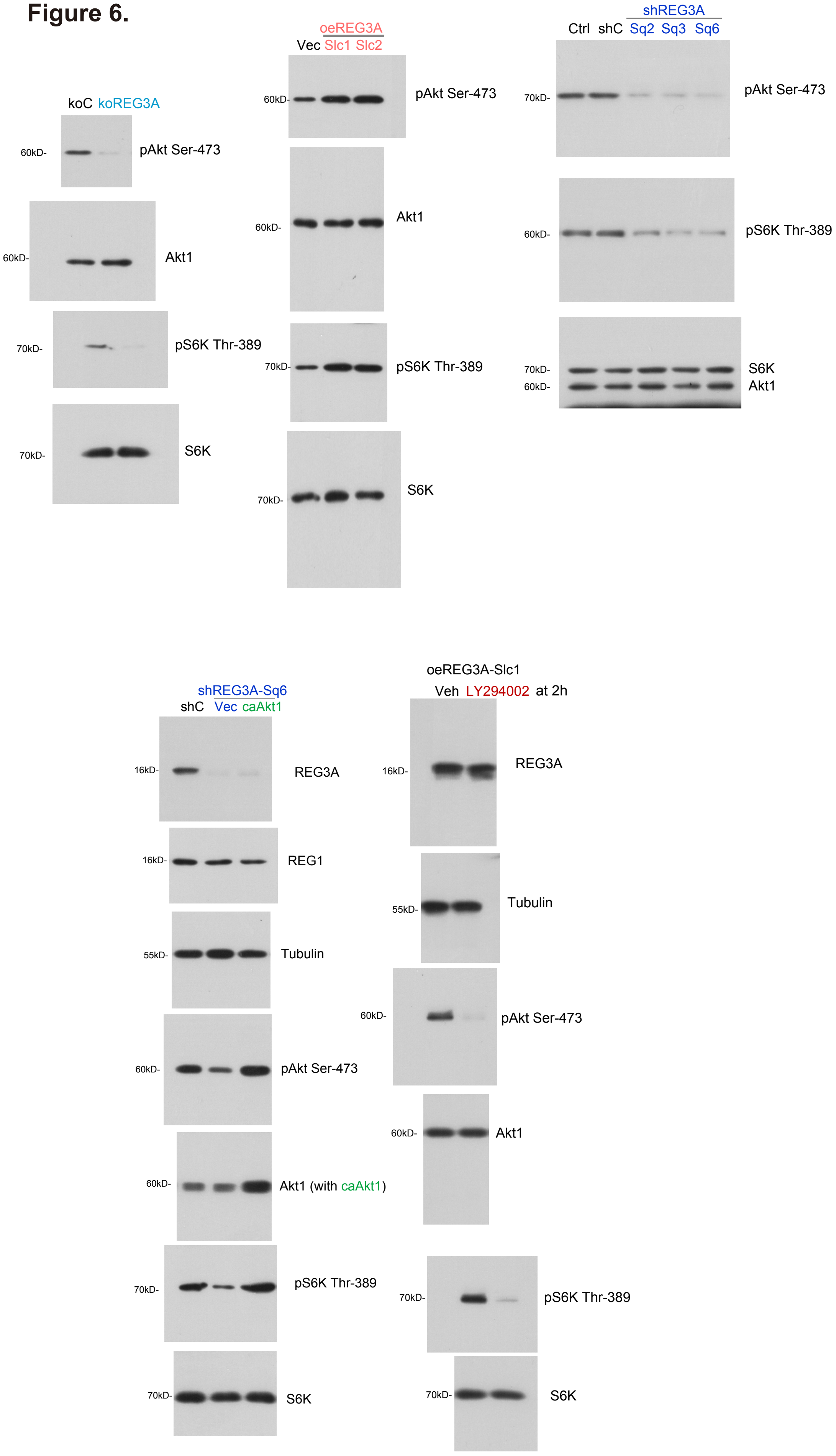

koC koREG3A
60kD-
pAkt Ser-473
60kD-
Akt1
70kD-
pS6K Thr-389
70kD-
S6K
oeREG3A
Vec Slc1 Slc2
60kD-
pAkt Ser-473
60kD-
Akt1
70kD-
pS6K Thr-389
70kD-
S6K
shREG3A
Ctrl shC Sq2 Sq3 Sq6
70kD-
pAkt Ser-473
60kD-
pS6K Thr-389
70kD-
60kD-
S6K
Akt1
shREG3A-Sq6
shC Vec caAkt1
16kD-
REG3A
16kD-
REG1
55kD-
Tubulin
60kD-
pAkt Ser-473
60kD-
Akt1 (with caAkt1)
70kD-
pS6K Thr-389
70kD-
S6K
oeREG3A-Slc1
Veh LY294002 at 2h
16kD-
REG3A
55kD-
Tubulin
60kD-
pAkt Ser-473
60kD-
Akt1
70kD-
pS6K Thr-389
70kD-
S6K

Figure 8.

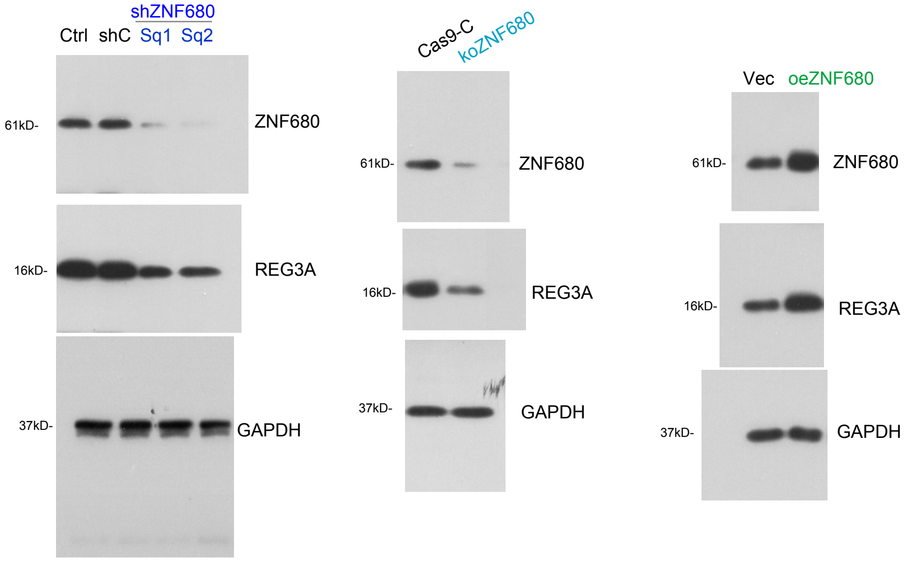

Figure 9.

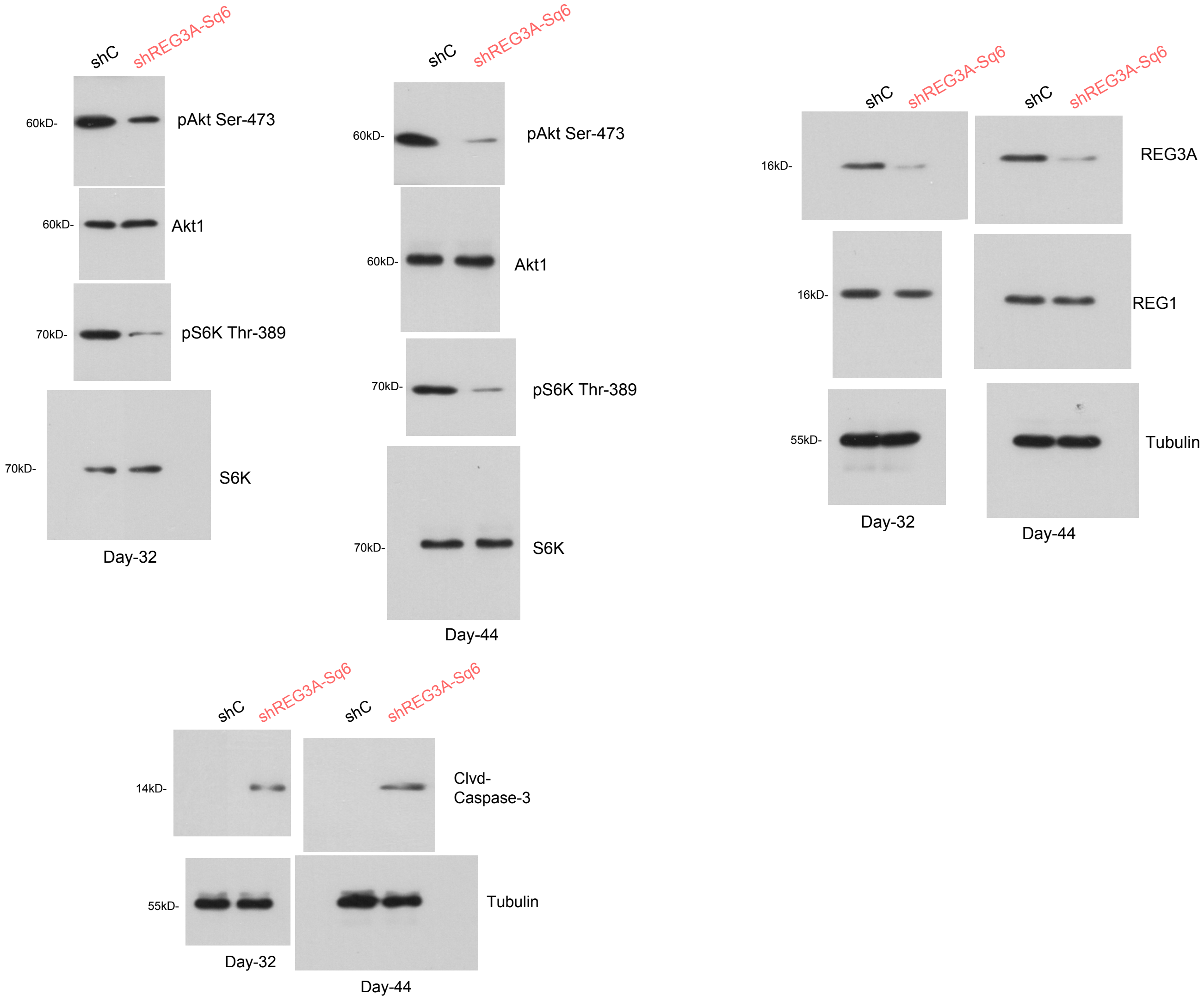

Supplement: Supplementary file 1 — Supplementary Material 1 [file 13058_2024_1845_MOESM1_ESM.pdf]
